# Molecular characterization of *Lipoptena cervi* from environmental samples collected in Poland

**DOI:** 10.1016/j.ijppaw.2020.12.005

**Published:** 2020-12-25

**Authors:** Remigiusz Gałęcki, Jerzy Jaroszewski, Tadeusz Bakuła, Xuenan Xuan

**Affiliations:** a Department of Veterinary Prevention and Feed Hygiene, Faculty of Veterinary Medicine, University of Warmia and Mazury in Olsztyn, Oczapowskiego 13, 10-719, Olsztyn, Poland; b Department of Pharmacology and Toxicology, Faculty of Veterinary Medicine, University of Warmia and Mazury in Olsztyn, Oczapowskiego 13, 10-719, Olsztyn, Poland; c National Research Center for Protozoan Diseases, Obihiro University of Agriculture and Veterinary Medicine, Obihiro, Hokkaido, 080-8555, Japan

**Keywords:** 16S rRNA gene, deer keds, Ectoparasite, Hippoboscidae, Louse flies, Phylogeny, PCR, Sequencing

## Abstract

The activity of *Lipoptena cervi* has intensified in Poland in recent years. The population genetics of this ectoparasite in Poland has never been described in the literature. The objectives of this study were to investigate the population genetics of *L. cervi* in selected regions of Poland, to evaluate molecular differences between *L. cervi* populations, and to determine phylogenetic relationships with other *L. cervi* sequences obtained in previous studies. In 2019, louse flies were sampled in natural mixed forests in five Polish voivodeships. Seven samples of *L. cervi* were collected from each voivodeship, and a total of 35 insects were analyzed molecularly. In the first step, *Lipoptena* spp. were identified to species level under a stereoscopic microscope. A fragment of the rRNA 16S gene was used as a marker to identify *L. cervi* by the PCR assay. The sequences were assigned accession numbers MT337409 to MT337416. A total of eight haplotypes were identified, two of which were dominant. In the obtained sequences, intraspecific pairwise genetic distances varied between 0.000 and 0.0496 (*m* = 0.0135; *SD* = 0.0149; *SE* = 0.0006; *V* = 110.11). Mean interpopulation diversity was d = 0.0135 (*SE* = 0.0027). The acquired nucleotide sequences were highly similar to the sequences from the Czech Republic (MF495940, AF322437), Lithuania (MN889542-MN889544) and Poland (MF541726–MF541729). The similarity with GenBank sequences ranged from 97.24% to 100%. This study revealed two dominant haplotypes of *L. cervi* in Poland, MT337410 and MT337413. Fragments of the analyzed sequences were detected in only one voivodeship. These findings suggest that the two dominant sequences are the oldest sequences that gave rise to the locally identified haplotypes. The lack of significant correlations with the sequences obtained in regions situated west of the research sites suggests the presence of other genetic populations in Europe.

## Highlights

• Eight new haplotypes with 0.000–0.0496 intraspecific pairwise genetic distance.
• Similarity with sequences from GenBank ranged from 97.24% to 100%.
• Two dominant haplotypes of *L.cervi* were observed in Central Europe.
• Part of the sequences have been discovered in single studied location.
• The lack of significant connections with sequences obtained from West Europe.

## Graphical abstract

**Figure:**
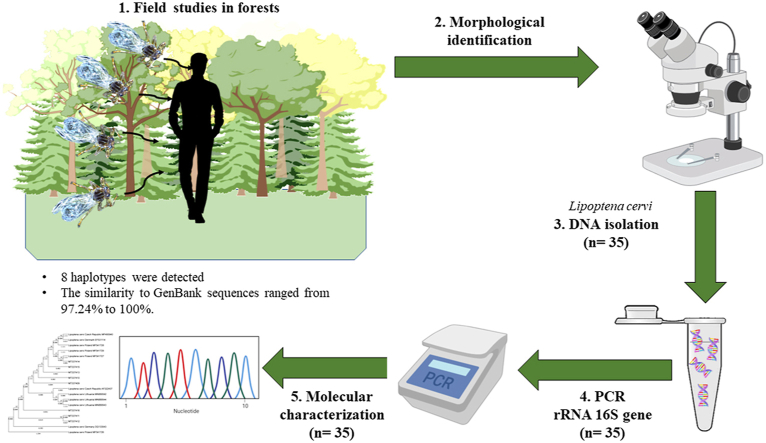

## 1 Introduction

The population of wild ungulates has been increasing steadily in Poland. The high increase in the number of cervids is particularly noteworthy (Zalewski et al. 2018). These phenomena also affect the populations of interacting species, especially ectoparasites (Madslien et al., 2012). Deer keds are parasites with a narrow host range, and they are directly associated with cervids (Härkönen et al., 2010). The activity of *Lipoptena* spp. has intensified in recent years, particularly in forests (Gałęcki et al. 2020). Keds are obligatory hematophagous ectoparasites of birds and mammals which reproduce by adenotrophic viviparity. The genus *Lipoptena* spp. belongs to the family Hippoboscidae and consists of 32 species (Dick, 2006). The species important for veterinary medicine include *Lipoptena capreoli*, *Lipoptena cervi, Lipoptena depressa*, *Lipoptena fortisetosa, Lipoptena mazamae* and *Neolipoptena ferrisi.* When keds find a definitive host, their wings are broken off at the base, leaving behind a stump (Lloyd, 2009).

*Lipoptena cervi* is an indigenous species of the Palearctic region. Its presence in Central and Northern Europe has been described in detail. *Lipoptena cervi* populations have been reported from Algeria, Great Britain and northern China and the Eastern United States (Skvarla and Machtinger, 2019). The northern distribution limit currently lies at approximately 65°N, and it is gradually moving northwards (Hackman, 1979; Hackman et al., 1983). *Lipoptena cervi* colonizes only wild ruminants which are specific host species for that ectoparasite. This animals also play an important role in the spread of *L. cervi* because keds can cover a distance of up to 50 m in the search for a host (Paakkonen, 2012). The life cycle of louse flies lasts up to 270–370 days with winter diapause in Europe, and it is determined mainly by climate conditions (Borowiec, 1984; McAlpine, 1987). Adult flies need to find a host soon after emergence to survive and breed. Host-seeking flies are typically observed between August and October (Hackman et al., 1983) when high temperatures prevail.

The prevalence and severity of *L. cervi* infestations can be extremely high in cervids. In Poland, this ectoparasite has been detected in numerous cervids and other animals. Deer keds were identified in 64% of European roe deer (Kadulski, 1996), 76% of fallow deer, and 78% of European red deer (Szczurek and Kadulski, 2004). Louse flies were also found to colonize European bison (Izdebska, 2001). The presence of *L. cervi* was noted in non-specific hosts such as companion animals (Sokół and Gałęcki, 2017), as well as in livestock (Dehio et al., 2004). One host is colonized by 9.9 flies on average (Szczurek and Kadulski, 2004). However, Vikøren et al. (2008) found more than 16,000 deer keds in a single moose. In specific hosts, massive colonization by *L. cervi* can lead to severe alopecia (Madslien et al., 2011). Most animals infested by *L. cervi* develop skin lesions, including acute to chronic, multifocal to coalescing, and eosinophilic to lymphocytic dermatitis (Madslien et al., 2011). The presence of L. cervi on the Tyrolean Ötzi mummy dating back more than 5000 years suggests that this species was also a human parasite in the past (Gothe and Schöl, 1994). Nowadays, the incidental infestation of humans with *L. cervi* has also been reported (Härkönen et al., 2009; Kortet et al., 2010). Humans bitten by louse flies can develop dermatitis, allergic rhinoconjunctivitis or even anaphylactic shock (Rantanen et al., 1982; Laukkanen et al., 2005; Decastello and Farkas, 2010). According to Härkönen et al. (2009), the number of people who require medical treatment for deer ked dermatitis will continue to increase. *Lipoptena cervi* can also serve as a vector for the transmission of several pathogens, including *Anaplasma* spp., *Bartonella* spp. and *Trypanosoma* spp. (Böse and Petersen, 1991; Hornok et al., 2011; De Bruin et al., 2015).

Most research into *L. cervi* has been carried out in Fennoscandia. This ectoparasite has attracted little interest from Central European researchers after 2000. In Poland, research into *L. cervi* was discontinued, and advanced research techniques, such as molecular biology tools, have been used on a small scale only (Szewczyk et al., 2017). *Lipoptena cervi* is unable to cross long distances, and it may form isolated populations when cervid migrations occur on a small scale. Trout et al. (2010) observed certain similarities between *Lipoptena* spp. and *Glossina* spp., including low reproductive rates and habitat limitations due to host preferences, and concluded that *Lipoptena* spp. populations should be characterized by pronounced genetic variation. In Poland, there is a general scarcity of evidence for genetic variation within *L. cervi* populations in distinct niches. Kurina et al. (2019) have argued that the precise identification, distribution and bionomics of *L. cervi* are of utmost importance for animal and human health, and imperative for vector control. Research into *L. cervi* seems to be justified due to the potential migration of host species between countries. A better understanding of deer ked haplotypes in Central Europe would support the identification of potential pathogens that are transmitted between migrating deer populations.

In view of the above, the objectives of this study were to investigate the population genetics of *L. cervi*, to evaluate molecular differences between *L. cervi* populations, and to determine phylogenetic relationships with other *L. cervi* sequences obtained in previous studies.

## 2 Materials and methods

### 2.1 Collection of deer ked samples

**Fig. 1:**
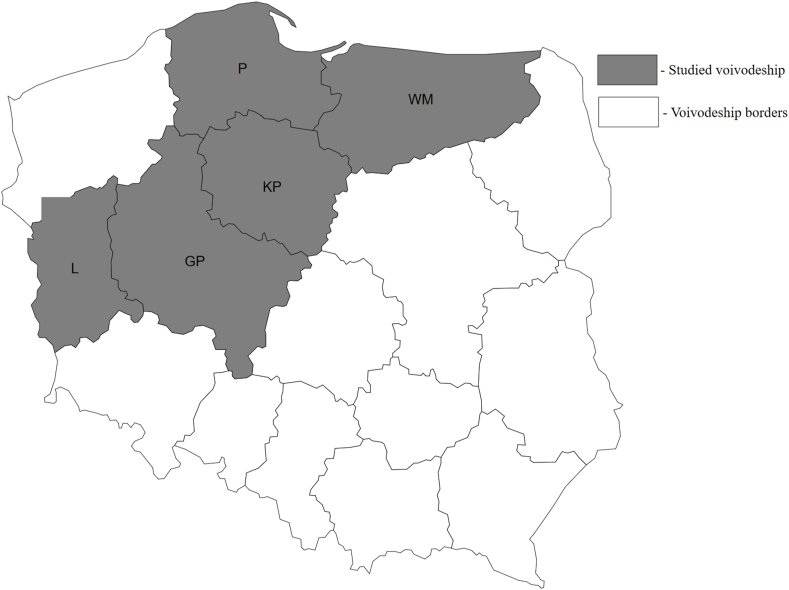
Map of Poland with highlighted voivodships where *Lipoptena cervi* samples were collected. Legend: GP - Greater Poland voivodeship; KP - Kuyavia-Pomerania voivodeship; L - Lubusz voivodeship; P - Pomerania voivodeship; WM - Warmia-Masuria voivodeship.

Louse flies were collected in 2019 in natural mixed forests in Poland. The study was conducted in five voivodeships: Greater Poland, Kuyavia-Pomerania, Lubusz, Pomerania, and Warmia-Masuria. A detailed map of the surveyed regions is presented in Fig. 1. *Lipoptena cervi* imagines were sampled from randomly selected forest complexes in each voivodeship. Scots pine was the predominant tree species in the studied areas. The underwood and undergrowth consisted of plant species typical of the Polish climate and geographic zone. Fresh traces of cervid, including red deer and European roe deer, were found in each location. Keds were collected by the investigators during walks in forests. The investigators wore brown cotton clothing covering the entire body, and they collected samples immediately after keds had landed on clothing. The obtained samples were placed in separate test tubes filled with 70% ethanol. Seven samples of *L. cervi* were collected from each voivodeship for further analyses.

### 2.2 Species identification

The collected samples were transported to the Biological Hazard Laboratory at the Faculty of Veterinary Medicine of the University of Warmia and Mazury in Olsztyn. Ked species was identified based on morphological characteristics under the Leica M165C stereoscopic microscope (Leica, Wetzlar, Germany). Measurements were carried out with the use of Leica Application Suite 4.4 (Leica, Wetzlar, Germany). Deer keds were identified to species level based on body dimensions, wing venation, length and structure of palpi, and the number of erect hairs on the mesonotum (Borowiec, 1984; Maa, 1969; Andreani et al., 2019).

### 2.3 DNA extraction

Seven *L. cervi* samples (n = 35) from each voivodeship were randomly selected for molecular analyses. In the first step, keds were air-dried for 15 min and then individually crushed with a sterile rod in sterile Eppendorf tubes. Genomic DNA was extracted from each specimen with the Sherlock AX universal kit (A&A Biotechnology, Gdynia, Poland) according to the manufacturer's instructions. DNA was eluted in 40 μL of TE buffer, and the concentration of the extracts was checked using the Nano Drop 2000 spectrophotometer (Thermo Fisher Scientific, Waltham, USA). The extracted DNA was stored at −20 °C until analysis.

### 2.4 PCR reaction

A fragment of the mitochondrial 16S rRNA gene of *Lipoptena* spp. with an estimated length of 412 bp was amplified by PCR. The PCR primers designed by Szewczyk et al. (2017) were used in amplification: L700F (5′-AAAGTTTAACCTGCCCACTGAT-3′) and L1213R (5′- CTGAACTCAGATCACGTAAGAAT -3′). The following cycling conditions were applied: initial denaturation at 92 °C for 3 min, 35 cycles of denaturation at 95 °C for 10 s, annealing at 60 °C for 10 s, extension at 68 °C for 30 s, followed by final extension at 68 °C for 5 min (Szewczyk et al. 2017). Each reaction was performed in a final volume of 25 μl containing 2.5 μl of 10X Standard Taq Reaction Buffer (Biolabs, USA), 0.5 μl of 10 mM dNTPs (Biolabs, USA), 0.5 μl of 10 μM of each primer, 1 μl of *L. cervi* DNA template, 0.125 μl of Taq DNA Polymerase (Biolabs, USA), and 19.875 μl of double distilled water as a negative control. The PCR products were electrophoresed on a 2% agarose gel, stained with ethidium bromide and viewed under a UV transilluminator.

### 2.5 Sequencing

After the PCR reaction, DNA samples were purified by the ethanol precipitation method described by Weinberger (2000). Using a previously described primer set (L700F/L1213R), cycle sequencing was performed with the BigDye Terminator Cycle Sequencing Kit (Applied Biosystems, Foster City, CA, USA), and the results were analyzed with the ABI PRISM 3100 Genetic Analyzer (Applied Biosystems, Foster City, CA, USA). The obtained nucleotide sequences were edited in BioEdit software (Hall, 1999) and compared with the data registered in the GenBank database using the BLAST-NCBI program. Phylogenetic analyses of the obtained rRNA 16S gene sequences and homologous GenBank sequences were performed using the maximum likelihood method in MEGA 10.1.17 (https://www.megasoftware.net) Bootstrap confidence values for branching reliability were calculated in 10,000 replicates. The sequences obtained from GenBank are listed in Table 1.

**Table 1:** Sequences used in the phylogenetic analysis.

Sequence ID	Species	Country of isolation	Reference
MT337409- MT337416	*Lipoptena cervi*	Poland	Current study
MF541726–MF541729*	*Lipoptena cervi*	Poland	Szewczyk et al. (2017)
MF495940	*Lipoptena cervi*	Czech Republic	Sochova et al. (2017)
AF322437	*Lipoptena cervi*	Czech Republic	Nirmala et al. (2001)
DQ133043	*Lipoptena cervi*	Germany	Dittmar et al. (2006)
MN889542- MN889544	*Lipoptena cervi*	Lithuania	Radzijevskaja et al. (2020)
EF531114	*Lipoptena cervi*	Denmark	Petersen et al. (2007)

Legend: *- sequences obtained in studied locations (Warmia-Masuria voivodeship).

### 2.6 Nucleotide sequence accession numbers

The representative sequences of *L. cervi* obtained in this study were deposited in the GenBank database of the National Center for Biotechnology Information. The sequences were assigned accession numbers MT337409 to MT337416.

### 2.7 Statistical analysis

Descriptive statistics, including the mean (*m*), standard deviation (*SD*) and variation (*V*), were calculated in the Statistica 13.3 program (TIBCO Software Inc., Palo Alto, USA). Intraspecific pairwise genetic distances, mean interpopulation diversity, mean genetic distances, standard error (*SE*), mean evolutionary distance, and nucleotide frequencies were calculated in MEGA 10.1.17 (https://www.megasoftware.net). Bootstrap confidence intervals were calculated in 10,000 replicates.

## 3 Results

Three haplotypes (MT337410, MT337413, MT337416) were detected in the Greater Poland voivodeship. Four haplotypes were found in the Kuyavia-Pomerania voivodeship (MT337409, MT337410, MT337413, MT337415). Three haplotypes were acquired in the Lubusz voivodeship (MT337410, MT337412, MT337413). Three haplotypes were identified in the Pomerania voivodeship (MT337410, MT337413, MT337414), and two (MT337410, MT337411) – in the Warmia-Masuria voivodeship. The detailed distribution of haplotypes in the examined locations is presented in Table 2.

**Table 2:** Molecular characterization of *Lipoptena cervi* in the studied sites.

No. of *Lipoptena cervi* haplopypes in examided voivodeships					
Detected haplotypes	Greater Poland (n = 7)	Kuyavia-Pomerania (n = 7)	Lubusz (n = 7)	Pomerania (n = 7)	Warmia-Masuria (n = 7)
MT337409	–	1	–	–	–
MT337410	4	3	3	3	5
MT337411	–	–	–	–	2
MT337412	–	–	2	–	–
MT337413	2	1	2	3	–
MT337414	–	–	–	1	–
MT337415	–	2	–	–	–
MT337416	1	–	–	–	–

In the obtained sequences, intraspecific pairwise genetic distances varied between 0.000 and 0.0496 (*m* = 0.0135; *SD* = 0.0149; *SE* = 0.0006; *V* = 110.11). Mean interpopulation diversity was d = 0.0135 (*SE* = 0.0027). Mean genetic distances within voivodeships were: d = 0.013 (*SE* = 0.003) for the Greater Poland voivodeship; d = 0.004 (*SE* = 0.002) for the Kuyavia-Pomerania voivodeship; d = 0.018 (*SE* = 0.004) for the Lubusz voivodeship; d = 0.021 (*SE* = 0.005) for the Pomerania voivodeship; and d = 0.007 (*SE* = 0.003) for the Warmia-Masuria voivodeship. Mean genetic distances between voivodeships are presented in Table 3. The mean evolutionary rates in these categories were 0.00, 0.00, 0.00, 0.03 and 4.97 substitutions per site. Nucleotide frequencies were determined at: A = 37.55%, T = 38.82%, C = 9.54%, and G = 14.10%.

**Table 3:** Mean genetic distances of *Lipoptena cervi* between voivodeships.

Studied voivodeships	GP	Mean genetic distance	(d)	P	WM
		KP	L		
GP (n = 7)	–	–	–	–	–
KP (n = 7)	0.0083	–	–	–	–
L (n = 7)	0.0159	0.0125	–	–	–
P (n = 7)	0.0199	0.0165	0.0174	–	–
WM (n = 7)	0.0099	0.0062	0.0136	0.0169	–

Legend: GP - Greater Poland voivodeship; KP - Kuyavia-Pomerania voivodeship; L - Lubusz voivodeship; P - Pomerania voivodeship; WM - Warmia-Masuria voivodeship; n - number of samples.

**Fig. 2:**
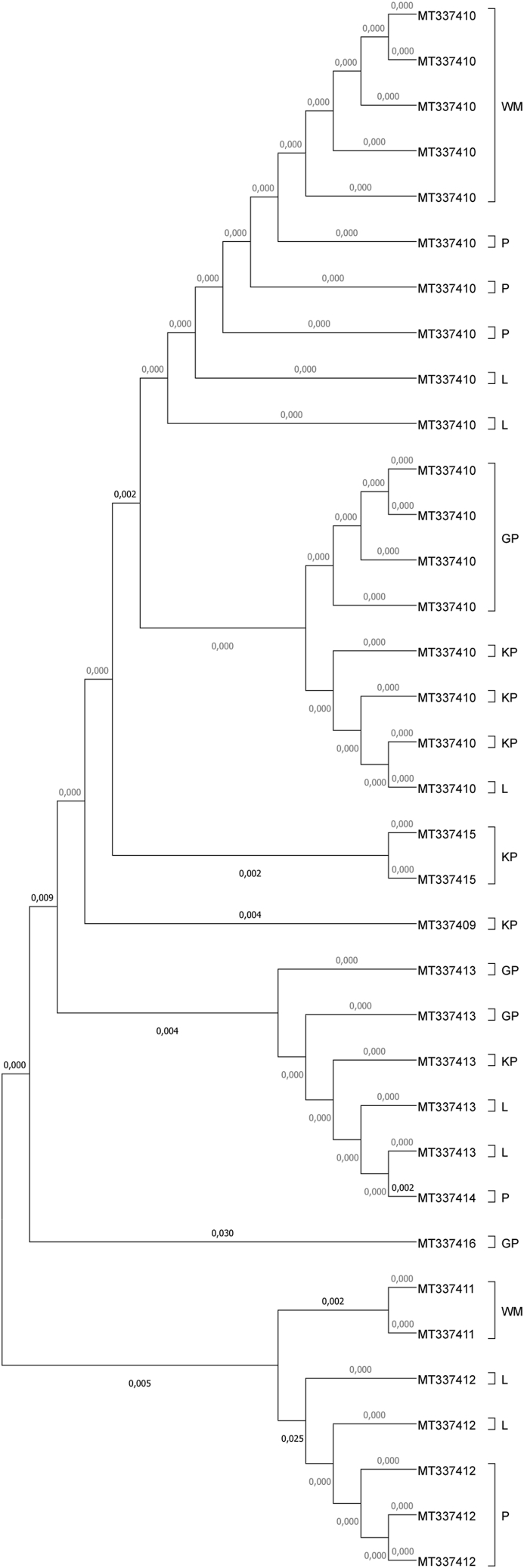
Phylogenetic analysis of the rRNA 16S gene in the examined sequences of *Lipoptena cervi* from the studied voivodships. Legend: Phylogenetic topology for the maximum likelihood analysis of the partial rRNA 16S gene sequence of *Lipoptena cervi*. The unique haplotypes identified in the study are labeled with the corresponding sequence identification numbers. Bootstrap confidence values for branching reliability were calculated in 10,000 replicates. G - Greater Poland voivodeship; KP - Kuyavia-Pomerania voivodeship; L - Lubusz voivodeship; P - Pomerania voivodeship; WM - Warmia-Masuria voivodeship.

**Fig. 3:**
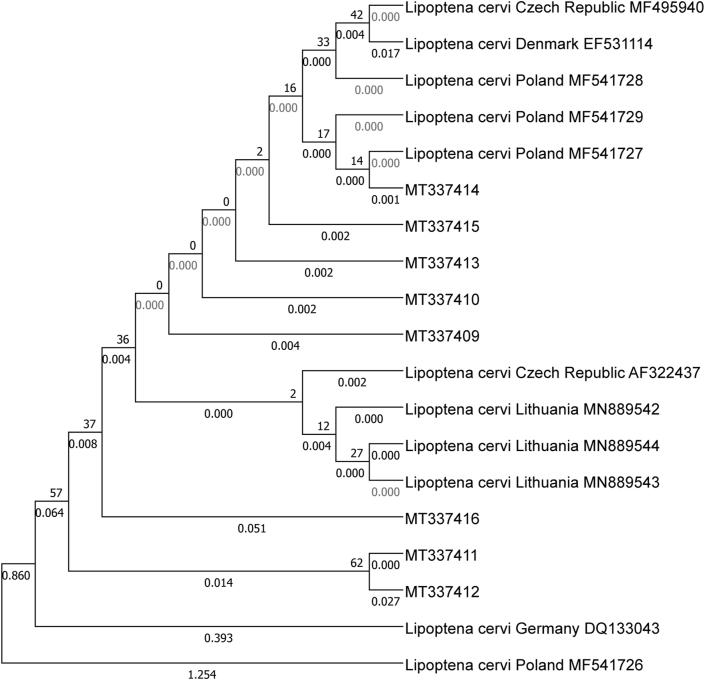
Phylogenetic analysis of the rRNA 16S gene in *Lipoptena cervi* relative to other sequences. Legend: Phylogenetic topology for the maximum likelihood analysis of the partial rRNA 16S gene sequence of *Lipoptena cervi*. The unique haplotypes identified in this study are labeled with the corresponding sequence identification numbers. The reference sequences available in GenBank are indicated in the tree. Bootstrap confidence values for branching reliability were calculated in 10,000 replicates.

Eight sequences (MT337409- MT337416) were characterized by 97.24%% to 100% similarity with *L. cervi* sequences from the Poland and Lithuania (MF541726, MF541729, MN889544, MN889544). No similarities were found between the identified sequences and the haplotypes from Denmark and Germany (Dittmar et al., 2006; Petersen et al., 2007). Detailed data, including the ID of the closest match, country of origin, and percent sequence identity, are presented in Table 4. Phylogenetic trees are presented in Fig. 2, Fig. 3.

**Table 4:** Comparison of the acquired sequences with GenBank sequences.

Sequence ID	Closest match ID	Country of origin	Percentage match	Reference
MT337409	MF541726	Poland	99.51%	Szewczyk et al. (2017) Radzijevskaja et al. (2020)
	MN889542	Lithuania	99.58%	
MT337410	MF541726	Poland	100%	
	MN889544	Lithuania	100%	
MT337411	MF541729	Poland	99.51%	
	MN889544	Lithuania	99.36%	
MT337412	MF541726	Poland	97.46%	
	MN889542	Lithuania	97.24%	
MT337413	MF541726	Poland	100%	
	MN889542	Lithuania	99.79%	
MT337414	MF541726	Poland	100%	
	MN889542	Lithuania	99.79%	
MT337415	MF541726	Poland	100%	
	MN889542	Lithuania	99.79%	
MT337416	MF541726	Poland	98.06%	
	MN889544	Lithuania	98.01%

## 4 Discussion

This study revealed two dominant haplotypes of *L. cervi* (MT337410 and MT337413) in Poland. These haplotypes were found in nearly all studied regions. Some sequences were detected in only one voivodeship. These findings suggest that the two dominant sequences are the oldest sequences that gave rise to the locally identified haplotypes. These haplotypes probably spread throughout Poland and evolved independently, by natural selection, into the sequences obtained in this study. This observation is supported by the differences in the analyzed gene. The expansion of louse flies is boundless in the temperate climate (due to the potential migration of cervids); therefore, the population and diversity of keds could be nearly identical in the studied locations (despite individual mutations). New local haplotypes are likely to be discovered in the future due to low reproductive rates and habitat limitations resulting from host preferences (Trout et al., 2010). It should also be noted that the genetic diversity of the *L. cervi* population in Central Europe suggests the presence of common ancestors and continuous migrations (gene flows) across countries. The above could also be attributed to limited panmixia because reproduction occurs only among individuals colonizing the same host. The presence of stable L. cervi populations in some areas probably induced genetic selection that was influenced by environmental factors and the availability of host species. The above suggests that *L. cervi* easily adapt to new ecosystems. Very close similarities were observed between the studied sequences of *L. cervi* and the sequences from the Czech Republic, Lithuania and Poland deposited in GenBank (Nirmala et al., 2001; Szewczyk et al., 2017; Sochova et al., 2017; Radzijevskaja et al., 2020). Interestingly, the sequences from Germany and Denmark (Dittmar et al., 2006; Petersen et al., 2007) did not match the current results, which could point to the presence of genetically different populations in Europe. However, only several 16S marker sequences of the studied ectoparasite are available in GenBank, and few haplotypes are geographically limited.

Molecular differences have been observed between *Lipoptena* spp. species despite the fact that the 16S rRNA gene is relatively resistant to genetic variation. Genetic variation was reported by Trout et al. (2010) in a study of *Lipoptena mazamae*. They identified six unique haplotypes by amplifying 16S markers in the PCR assay. Similar observations have been made in other ectoparasites, including bed bugs, tsetse flies and ticks (Krafsur et al., 2000; Szalanski et al., 2008; Kulakova et al., 2014; Livanova et al., 2015). These species as well as *L. cervi* are characterized by considerable population fragmentation, which can lead to isolation and genetic aberration. For this reason, the use of the 16S rRNA gene in analyses of genetic diversity within limited populations seems to be justified. In the future, the results of this study should be validated by analyzing genetic variation in the cytochrome c oxidase I (COX1) gene of *L. cervi* in Poland. An analysis of the correlations between COX1 and 16S rRNA gene sequences could also deliver new insights.

Population studies seem to be relevant because *L. cervi* is an underestimated vector of veterinary importance. This parasite is not fully understood from the epidemiological point of view. The potential flow of pathogens across regions could be predicted be examining the results of population genetics studies and the transfer of infectious agents by selected haplotypes. A thorough knowledge of the genetic profile of *L. cervi* combined with pathogen detection would support the identification of potential high-risk areas. Further research is needed due to the steady increase in *L. cervi* population. The presence of *L. cervi* on non-specific hosts or in non-specific locations (such as cities) could also indicate that the species rapidly assimilates to new environmental conditions. The above could be correlated with specific haplotypes that better adapt to non-specific ecosystems. Migratory processes could contribute to the spread of new pathogens to previously uncolonized areas, including human settlements. Previous studies of Hippoboscidae confirmed that pathogens could be horizontally transmitted. Bacteria of the genera *Bartonella* spp.*, Borrelia* spp. and *Coxiella*-like bacteria have been identified in *Lipoptena* spp., but the ectoparasite's ability to transmit pathogens has been confirmed only for *Bartonella* spp. (Doby et al., 1994; Hulinsky et al., 2002; Hornok et al., 2011; De Bruin et al., 2015; Korhonen et al., 2015; Lee et al., 2016). Protozoa such as *Anaplasma phagocytophilum* and *A. ovis*, *Theileria luwenshuni*, *T. ovis* and *Trypanosoma* spp. have also been found in these insects (Böse and Petersen, 1991; Hornok et al., 2011; Víchová et al., 2011; Lee et al., 2016). However, this problem requires more detailed research.

The population of *L. cervi* can be expected to rise in Central Europe due to climate change and an increase in host populations. According to Härkönen et al. (2010), the increase in winter and summer temperatures associated with global warming enhances the performance of *L. cervi* and prolongs its flight period. In the studied regions, high winter and summer temperatures could potentially intensify the migration of this ectoparasite species to human settlements. However, further research is needed to confirm this assumption. Higher temperatures could significantly affect the parasite's developmental cycle by shortening winter diapause, altering the phenology of *L. cervi,* and increasing its population due to lower mortality in winter. The steady rise in the deer population in Poland also increases the availability of feed for deer keds. In order to survive, newly-emerged imagines of *L. cervi* have to rapidly find a host. This ectoparasite travels short distances (up to 50 m) in the search of a host, which is why an increase in the density of the wild ungulate population can also reduce mortality associated with the lack of hosts. Cervid migrations play a significant role in the spread of *L. cervi*. This ectoparasite can survive for more than a year on the host (Härkönen et al. 2010); therefore, the new generation may develop even in distant locations. In this study, the above was observed in the two most commonly detected haplotypes. It should also be noted that the related species of *L. fortisetosa* has been identified in Central Europe (Andreani et al., 2019; Gałęcki et al., 2020). In the future, efforts should be made to differentiate between *L. cervi* and *L. fortisetosa,* and to explore potential hybridization between these two closely related species. Due to the apparent physiological similarities and similar activity patterns of *L. cervi* and *L. fortisetosa* (Oboňa et al., 2019) incorrect identification could produce misleading results in future research.

## 5 Conclusions

The present study reveal genetic variation in the 16S rRNA gene of the *L. cervi* population in selected Polish voivodeships. The studied regions were colonized by two dominant haplotypes of *L. cervi* that have been identified in other Central European countries, and local haplotypes were detected in individual cases. The lack of significant correlations with the sequences obtained in regions situated west of the research sites suggests the presence of other genetic populations in Europe. In the future, the population genetics of *L. cervi* should be investigated in greater detail to contribute to the development of preventive programs aiming to reduce ectoparasitic populations in the environment and minimize the negative impact of *L. cervi* on wild animals, livestock and humans.

## Funding

Project financially co-supported by the 10.13039/501100004569Minister of Science and Higher Education under the program entitled "Regional initiative of Excellence" for the years 2019–2022, Project No. 010/RID/2018/19, amount of funding PLN 12,000,000. The funders had no role in study design, data collection and analysis, decision to publish, or preparation of the manuscript.

## Declaration of competing interestCOI

The authors declare that they have no conflict of interest.
